# A methodological quality assessment of systematic reviews and meta-analyses of antidepressants effect on low back pain using updated AMSTAR

**DOI:** 10.1186/s12874-020-0903-9

**Published:** 2020-01-23

**Authors:** Mohammad Hossein Panahi, Mostafa Mohseni, Razieh Bidhendi Yarandi, Fahimeh Ramezani Tehrani

**Affiliations:** 1 Department of Epidemiology, School of Public Health, Shahid Beheshti University of Medical Sciences, Tehran, Iran; 2 Neurosurgery Department, Shohada Tajrish Hospital, Shahid Beheshti University of Medical Sciences, Tehran, Iran; 3 Department of Epidemiology and Biostatistics, School of Public Health, Tehran University of Medical Sciences, Tehran, Iran; 4 Reproductive Endocrinology Research Center, Research Institute for Endocrine Sciences, Shahid Beheshti University of Medical Sciences, No 24, Parvane Street, Yaman Street, Velenjak, P.O.Box: 19395-4763, Tehran, Iran

**Keywords:** AMSTAR 2, Antidepressants, Low back pain, Meta-analysis, Systematic review

## Abstract

**Background:**

Antidepressants are prescribed widely to manage low back pain. There are a number of systematic reviews and meta-analyses which have investigated the efficacy of the treatments, while the methodological quality of them has not been assessed yet. This study aims to evaluate the methodological quality of the systematic reviews and meta-analyses investigating the effect of antidepressants on low back pain.

**Methods:**

A systematic search was conducted in PubMed, EMBASE, Medline, and Cochrane Library databases up to November 2018. The 16-item Assessment of Multiple Systematic Reviews (AMSTAR2) scale was used to assess the methodological quality of the studies. Systematic reviews and meta-analyses of the Antidepressants treatment effects on low back pain published in English language were included. There was no limitation on the type of Antidepressants drugs, clinical setting, and study population, while non-systematical reviews and qualitative and narrative reviews were excluded.

**Results:**

A total of 25 systematic reviews and meta-analyses were evaluated; the studies were reported between 1992 and 2017. Obtained results from AMSTAR2 showed that 11 (44%), 9 (36%) and 5 (20%) of the included studies had high, moderate and low qualities, respectively. 13(52%) of studies assessed risk of bias and 2(20%) of meta analyses considered publication bias. Also, 16 (64%) of the included reviews provided a satisfactory explanation for any heterogeneity observed in the results.

**Conclusions:**

Although the trend of publishing high quality papers in ADs effect on LBP increased recently, performing more high-quality SRs and MAs in this field with precise subgroups of the type of pains, the class of drugs and their dosages may give clear and more reliable evidence to help clinicians and policymakers.

## Introduction

Low back pain (LBP) is a major cause of disability. It was ranked first and sixth in terms of disability (YLDs) and overall burden (DALYs), respectively [1]. Pharmaceutical and non-pharmaceutical therapies are taken extensively to tackle this issue; in this way, guidelines provide a variety of suggested medicines and practices such as the use of nonsteroidal anti-inflammatory drugs (NSAIDs) and weak opioids in patients with non-specific/acute LBP for short periods [2–5]. Although antidepressants (ADs) are not recommended as the first-line prescribed medicine to manage LBP, they are taken widely [2, 6–9]. There is conflicting evidence about the effect of antidepressant, different studies showed their beneficial role in pain reduction while others have opposed them due to the high risk of adverse effects such as dry mouth, dizziness, nausea, headache, and constipation and no clear evidence of efficacy [10–13]. In addition, some systematic reviews (SRs) and meta-analyses (MAs) which summarized the results of the available evidence, provided heterogeneous results which make it difficult to decision regarding the efficacy of ADs [14–18].

An SR is a type of literature review which critically evaluates research studies. It can summarize results obtained from a plethora of studies helping researchers and clinicians to keep up with the new findings. MA is also a statistical approach to summarize the evidence extracted from secondary data obtained from the SR of studies in a specific subject. SRs and MAs provide a reference source for aiding experts in decision making. Despite their rapid growth and profound influence in health science, discrepancies of the results in studies on the same subject has made them unreliable in decision making. One reason is the matter of methodological quality of the reviews [19–21]. In this respect, evaluating the reliability and methodological quality of the studies is of great importance. There are some technical and methodological approaches to enrich SRs and MAs in order to reach valid results [22–26]. For this purpose, the Assessment of Multiple Systematic Reviews (AMSTAR) scale provides an appraisal tool for measuring the methodological quality of SRs [27, 28]. The purpose of this study was to assess the methodological quality of SRs and MAs of the role of ADs in treating LBP using the updated version of AMSTAR.

## Materials and methods

### Data sources and study selection

We searched for all SRs and MAs up to November 2018 using the PubMed, EMBASE, Medline, and Cochrane Library databases. Our search strategy followed the recommendations of the Cochrane Back Review Group [22–24]. Combinations of the following keywords were used in the search: “low back pain” AND “chronic low back pain” AND “non-specific low back pain” AND “sciatica” AND “leg pain” AND “antidepressant” AND (“TCA” OR “tricyclic antidepressants”) AND (“SSRI” OR “selective serotonin reuptake inhibitors”) AND (“SNRI” OR “serotonin and norepinephrine reuptake inhibitors”) AND (“TeCA” OR “tetracyclic antidepressants”) AND “meta-analysis” AND “systematic review”. The text words and MeSH terms were entered depending on the databases characteristics. The reference lists from retrieved articles were also screened for additional applicable studies.

### Inclusion and exclusion criteria

We included SRs and MAs of the ADs treatment effects on LBP published in English language. We also included all types of low back pain such as Chronic Low Back Pain (CLBP), Non-specific Low Back Pain (NLBP), Chronic Non-specific Low Back Pain (CNLBP) and sciatica, regardless of the cause of pain such as cancer, fracture, inflammatory disease, etc. There was no limitation on the type of ADs drugs, clinical setting, and study population, while non-systematical reviews and qualitative and narrative reviews were excluded.

### Study selection and data extraction

Screening of titles and abstracts of the retrieved studies for inclusion was conducted by two independent reviewers (RBY and MHP). The full texts of the eligible reviews were extracted and evaluated to determine whether they met the inclusion criteria by RBY and MHP. Any disagreements were resolved by consensus through discussion and the third person (FRT). For each study, the following information was extracted: authors, year of publication, study design, type of study and intervention, characteristics of study population, outcome measurement and summary of obtained 50 results. PRISMA flow diagram [29] was used to guide the process of inclusion and exclusion of studies.

### Assessment of methodological quality of included studies

Quality assessment was performed independently by two authors (RBY and MHP). Any discrepancies were resolved by discussion, and a blinded third reviewer was consulted if necessary. We used the updated Assessment of Multiple Systematic Reviews (AMSTAR2) appraisal tool to evaluate the methodological quality of eligible SRs and MAs [28]. It has some advantages compared to its previous version, such as the inclusion of non-randomized studies in SRs, and a different scoring system which helps reduce bias produced by quality scores obtained traditionally by summing up scores and getting an overall score [30]. AMSTAR2 contains 16 items; i.e., four domains have been added to this new version of AMSATR. Two of these were adopted directly from the ROBINS-I tool, namely, elaboration of the PICO and the way in which risk of bias was handled during evidence synthesis. Another one was the discussion of possible causes and significance of heterogeneity. The last new domain was the justification of selection of study designs to deal with non-randomized designs. The domain-specific questions in AMSTAR 2 are framed so that a “Yes” answer denotes a positive result. “Not Applicable” and “Cannot Answer” options in the original AMSTAR instrument were removed and “Partial Yes” responses have been provided where it is worthwhile to identify partial adherence to the standard. Moreover, the AMSTAR tool has a good agreement, reliability, construct validity, and feasibility to assess the quality of systematic reviews [31].

### Data analysis

Characteristics of the studies are reported in Table 1. In addition, Tables 2 and 3 show the results of AMSTAR2 domain (“Yes”, “Partial Yes”, “No”) of each included study. Moreover, the secular trend of the number and quality of included reviews was illustrated as well.

**Table 1:** Characteristics of included systematic reviews and meta analyses studies

	First author, Year, Country	Type of pain/ Outcome	Number/ Types of study included	Types of treatment	Results in terms of pain reduction/ Side effects
1	Riediger C, 2017 [12], Germany	CLBP/ Adverse events	Total:23, LBP:5/RCTs	*TCAs*: Amitriptyline, Desipramine, Nortriptyline, *SNRIs*: Venlafaxine, Milnacipran, Duloxetine, *TeCAs*: Mirtazapine, *SSRIs*: Fluoxetine	Higher risk for adverse effects compared to placebo, except nortriptyline.
	Onghena P, 1992 [32], Belgium	CLBP/ Pain	Total:39, LBP: 5 /RCTs	*TCAs*: Imipramine, Doxepin, Amitriptyline	Effective results in pain relief
2	Chung JWY, 2013 [18], China	CNLBP/ Pain, Global Improvement, Adverse events	Total:25, ADs:4/ RCTs	No specific subgroups	Statistically significant treatment effects in pain relief and side effects
3	Pinto RZ, 2012 [33], Australia	LBP, Sciatica/ Pain, Function	Total:23, ADs: 1/RCT	*TCAs*: Nortriptyline	No significant results in pain relief Data were insufficient
4	Urquhart DM, 2010 [34], Australia	NLBP/Pain, Function, Depression	10 RCTs	*TCAs* *SSRIs*	No clear evidence of effectiveness
5	Machado LAC, 2009 [35], Australia	CNLBP/ Pain	Total:74, AD_S_: 4/ RCTs	No specific subgroups	Small analgesic effect
6	Salerno SM, 2002 [36], USA	CLBP/ Pain, Function	9 RCTs	*TCAs*: Nortriptyline, Imipramine, Amitriptyline, *TeCAs*: Maprotiline, *SSRIs*: Paroxetine, *SNRIs*: Duloxetine, *Others*: Trazodone	Effective results in pain relief
8	Chou R, 2017 [11], USA	CLBP/ Pain, Function, Depression	Total:79, ADs: 16/1SR + 6RCTs	*TCAs* *SSRIs* *SNRIs*: Duloxetine	*SNRIs:* Effective on pain reduction *TCAs* and *SSRIs*: No significant results
9	Van Den Driest JJ, 2017 [17], The Netherlands	CLBP/Pain, Function, Adverse events	Total:7/ LBP: 4	*TCAs*: Amitriptyline vs. Pregabalin	Effective results in pain relief Similar side effect with comparator
10	National Guideline Centre (UK), 2016 [2],UK	LBP, Sciatica/ Pain, Function, Adverse events	Total:55, ADs: 10/RCTs	*TCAs*, *SSRIs*, *SNRIs*	No clear evidence of effectiveness. *SSRIs*, *SNRIs* significant adverse event
11	Chou R, 2016 [37], USA	CLBP/ Pain, Function	Total:153, ADs: 4 /1SR + 3 RCTs	*TCAs*, *SSRIs, SNRIs*: Duloxetine	*SSRIs and TCAs*: No effect on pain reduction *SNRIs:* small effect on pain reduction
12	Mercier A,2013 [38], France	NLBP, Sciatica/ Pain	Total:78, LBP:3/ RCTs	No specific subgroups	No AD treatments recommended. Only in the event of associated Depression
13	RomanoCL,2012 [39], Italy	CLBP/ Pain, Function, Depression	Total: 6, ADs: 1/ RCT	*TCAs*: Nortriptyline	No significant results for monotherapy
14	Morlion B, 2011 [40], Belgium	LBP/ pain, Function	Ads:10	No specific subgroups	Small benefits for Ads. *TCAs* are recommended
15	Kuijpers T, 2011 [16], The Netherlands	CNLBP/ Pain, Function, Adverse events	Total: 17, AD_S_: 5 / SR and MA	No specific subgroups	No clear evidence of effectiveness and side effects
16	Savigny P,2009 [41], UK	NLBP/ Pain, Function, Depression	1SR+ 10 RCTs	*TCAs*, *SSRIs*, *SNRIs*	No clear evidence of effectiveness
17	Chou R, 2007 [15], USA	CLBP/ Pain, Function, Adverse events	3 SR	*TCAs*: Nortriptyline, Imipramine, Amitriptyline, Desipramine *SSRIs:* Paroxetine *SNRIs*: Duloxetine, Venlafaxine *Others:* Maprotiline, Trazodone	Only *TCAs* have been shown effective. No evidence on *SNRIs, SSRIs.* Insufficient evidence for *Others.* Significantly higher risk for any adverse event.
18	Staiger THO, 2003 [42], USA	CLBP/ Pain, Function	7 RCTs	*TCAs*: Nortriptyline, Imipramine, Amitriptyline, *SSRIs:* Paroxetine, *TeCAs:* Maprotiline, *Others*: Trazodone	TCAs and TeCAs: moderate symptom reductions SSRIs: Not beneficial
19	White AP,2011 [43], USA	CLBP/ Pain, Function, Adverse events	Total: 6	*TCAs:* Desipramine, Imipramine *SSRIs:* Paroxetine, Fluoxetine	No effective than placebo. No differences between differing types of ADs.
20	Cawston H,2013 [44], France	CLBP/Pain	4 RCTs+ MAs	*SNRIs*: Duloxetine	No difference in efficacy between duloxetine and other oral pharmacological therapies.
21	Qaseem A, 2017 [45], USA	CLBP/ Pain	9 RCTs	*TCAs* *SSRIs* *SNRIs*: Duloxetine	No difference between *SSRIs* and *TCAs.* Duloxetine had small effect
22	Perrot S, 2006 [46], France	CLBP/ Pain	Total:99 4 on CLBP	*TCAs*: Nortriptyline, Imipramine, Amitriptyline, *TeCAs:* Maprotiline *SSRIs:* Paroxetine	*TCAs* and *TeCAs*: Moderate symptom reductions *SSRIs*: Not Beneficial
23	Perrot S, 2008 [47],France	CLBP/ Pain	Total:52 11 on CLBP	*TCAs*: Nortriptyline, Imipramine, Clomipramine, Amitriptyline *SSRIs:* Paroxetine, Bupropion *TeCAs:* Maprotiline *Others*: Trazodone	*SSRIs* seem to be less effective than *TCAs*
24	Patetsos E,2016 [48], Denmark	CLBP/ Pain	Total:36 2 on CLBP	*SSRIs*: Paroxetine	No significant results
25	Schnitzer Th J,2004 [49], USA	CLBP/ Pain	Total: 55, ADs: 7 RCTs	No specific subgroups	Evidence exists regarding the efficacy of antidepressants

*Abbreviations*: *CLBP* Chronic Low Back Pain, *NLBP* Non-specific Low Back Pain, *CNLBP* Chronic Non-specific Low Back Pain, *MA* Meta-Analysis, *R* Review, *NR* Narrative Review, *SR* Systematic Review, *CSR* Comprehensive Systematic Review, *ADs* Antidepressants, *TCAs* Tricyclic Antidepressants, *TeCA* Tetracyclic Antidepressant, *SSRIs* Selective Serotonin Reuptake Inhibitor, *SNRIs* Serotonin–Norepinephrine Reuptake Inhibitors, *SARI* Serotonin Antagonist and Reuptake Inhibitor

**Table 2:** Methodological quality of systematic reviews or meta-analyses using AMSTAR2

	First author	Type of study/Publication year	AMSTAR2 Quality Items																AMSTAR2 Classification
			1	2	3	4	5	6	7	8	9	10	11	12	13	14	15	16	
1	Onghena P	SR/MA 1992	Y	N	N	Y	N	N	Y	Y	PY	N	Y	N	N	N	N	N	Low
2	Salerno SM	SR/MA 2002	N	N	N	Y	Y	Y	Y	Y	PY	N	Y	PY	PY	N	Y	N	Moderate
3	Machado LAC	SR/MA 2009	Y	N	Y	Y	Y	Y	Y	Y	PY	N	Y	PY	PY	N	N	Y	Moderate
4	Urquhart DM	SR/MA 2010	Y	Y	Y	Y	Y	Y	Y	Y	Y	Y	Y	Y	Y	Y	Y	Y	High
5	White AP	SR/MA 2011	Y	N	Y	Y	Y	Y	Y	Y	Y	Y	N	Y	Y	Y	N	N	Moderate
6	Pinto RZ	SR/MA 2012	Y	N	Y	Y	Y	Y	Y	Y	Y	Y	Y	Y	Y	Y	N	Y	High
7	Cawston H	SR/MA 2013	Y	N	Y	Y	Y	Y	Y	Y	Y	Y	Y	Y	Y	Y	N	Y	High
8	Chung JWY	SR/MA 2013	Y	N	Y	Y	N	Y	Y	Y	Y	Y	Y	N	N	Y	N	Y	Moderate
9	Riediger C	SR/MA 2017	Y	N	Y	Y	N	N	Y	Y	Y	Y	Y	Y	Y	Y	N	Y	Moderate
10	Qaseem A	SR/MA 2017	Y	N	Y	Y	Y	Y	Y	Y	Y	Y	Y	Y	Y	Y	N	Y	High
11	Staiger THO	SR/2003	Y	N	Y	Y	Y	Y	Y	Y	PY	Y	NM	NM	Y	N	NM	N	Moderate
12	Schnitzer Th J	SR 2004	Y	N	Y	Y	Y	Y	Y	Y	PY	Y	NM	NM	PY	Y	NM	Y	Moderate
13	Perrot S	SR 2006	Y	N	N	Y	Y	Y	N	N	PY	N	NM	NM	PY	N	NM	N	Low
14	Chou R	SR/2007	Y	N	Y	Y	Y	Y	Y	Y	PY	Y	NM	NM	PY	Y	NM	Y	Moderate
15	Perrot S	SR 2008	Y	N	Y	Y	Y	Y	N	Y	PY	N	NM	NM	PY	N	NM	Y	Moderate
16	Savigny	SR/2009	Y	N	Y	Y	Y	Y	Y	N	Y	Y	NM	NM	Y	Y	NM	Y	High
17	Morlion B	SR/2011	N	N	Y	N	N	N	Y	Y	N	Y	NM	NM	N	N	NM	Y	Low
18	Kuijpers T	SR/2011	Y	N	Y	Y	Y	Y	Y	Y	Y	Y	NM	NM	Y	Y	NM	Y	High
19	Romano CL	SR/2012	Y	N	N	Y	Y	Y	Y	Y	N	Y	NM	NM	N	N	NM	Y	Low
20	Mercier A	SR/2013	Y	N	N	Y	Y	Y	Y	N	N	Y	NM	NM	N	N	NM	Y	Low
21	Patetsos E	SR 2016	Y	Y	Y	Y	Y	Y	Y	Y	Y	Y	NM	NM	Y	Y	NM	Y	High
22	Chou R	SR/2016	Y	Y	Y	Y	Y	Y	Y	Y	Y	Y	NM	NM	Y	Y	NM	Y	High
23	National Guideline	SR/2016	Y	Y	Y	Y	Y	Y	Y	Y	Y	Y	NM	NM	Y	Y	NM	Y	High
24	Chou R	SR/2017	Y	Y	Y	Y	Y	Y	Y	Y	Y	Y	NM	NM	Y	Y	NM	Y	High
25	Van Den Driest JJ	SR/2017	Y	N	Y	Y	Y	Y	Y	Y	Y	Y	NM	NM	Y	Y	NM	Y	High

*Y* Yes, *PY* Partial Yes, *N* No, *NM* No Meta-analysis

AMSTAR2 Classifications:

High: No or one non-critical weakness: the systematic review provides an accurate and comprehensive summary of the results of the available studies that address the question of interest

Moderate: More than one non-critical weakness: the systematic review has more than one weakness but no critical flaws. It may provide an accurate summary of the results of the available studies that were included in the review

Low: One critical flaw with or without non-critical weaknesses: the review has a critical flaw and may not provide an accurate and comprehensive summary of the available studies that address the question of interest

Critically low: More than one critical flaw with or without non-critical weaknesses: the review has more than one critical flaw and should not be relied on to provide an accurate and comprehensive summary of the available studies

**Table 3:** Methodological quality of the included meta-analyses and systematic reviews

Items		Y, n (%)	PY, n (%)	N, n (%)
1	Did the research questions and inclusion criteria for the review include the components of PICO (population, intervention, control group and outcome)?	23 (92)	0 (0)	2 (8)
2	Did the report of the review contain an explicit statement that the review methods were established prior to conduct of the review and did the report justify any significant deviations from the protocol?	4 (16)	0 (0)	21 (84)
3	Did the review authors explain their selection of the study designs for inclusion in the review?	20 (80)	0 (0)	5 (20)
4	Did the review authors use a comprehensive literature search strategy?	24 (96)	0 (0)	1 (4)
5	Did the review authors perform study selection in duplicate?	21 (84)	0 (0)	4 (16)
6	Did the review authors perform data extraction in duplicate?	22 (88)	0 (0)	3 (12)
7	Did the review authors provide a list of excluded studies and justify the exclusions?	22 (88)	0 (0)	3 (12)
8	Did the review authors describe the included studies in adequate detail?	22 (88)	0 (0)	3 (12)
9	Did the review authors use a satisfactory technique for assessing the risk of bias (RoB) in individual studies that were included in the review?	13 (52)	8 (32)	4 (16)
10	Did the review authors report on the sources of funding for the studies included in the review?	20 (80)	0 (0)	5 (20)
11	If meta-analysis (MA) was justified did the review authors use appropriate methods for statistical combination of results?	9 (90)	0 (0)	1 (10)
12	If meta-analysis was performed did the review authors assess the potential impact of RoB in individual studies on the results of the meta-analysis or other evidence synthesis?	5 (50)	2 (20)	3 (30)
13	Did the review authors account for RoB in individual studies when interpreting/ discussing the results of the review?	14 (56)	6 (24)	5 (20)
14	Did the review authors provide a satisfactory explanation for, and discussion of, any heterogeneity observed in the results of the review?	16 (64)	0 (0)	9 (36)
15	If they performed quantitative synthesis did the review authors carry out an adequate investigation of publication bias (small study bias) and discuss its likely impact on the results of the review?	2 (20)	0 (0)	8 (80)
16	Did the review authors report any potential sources of conflict of interest, including any funding they received for conducting the review?	20 (80)	0 (0)	5 (20)

*Y* Yes, *PY* Partial Yes, *N* No, *NM* No meta-analysis conducted

## Results

### Study identification

**Fig. 1:**
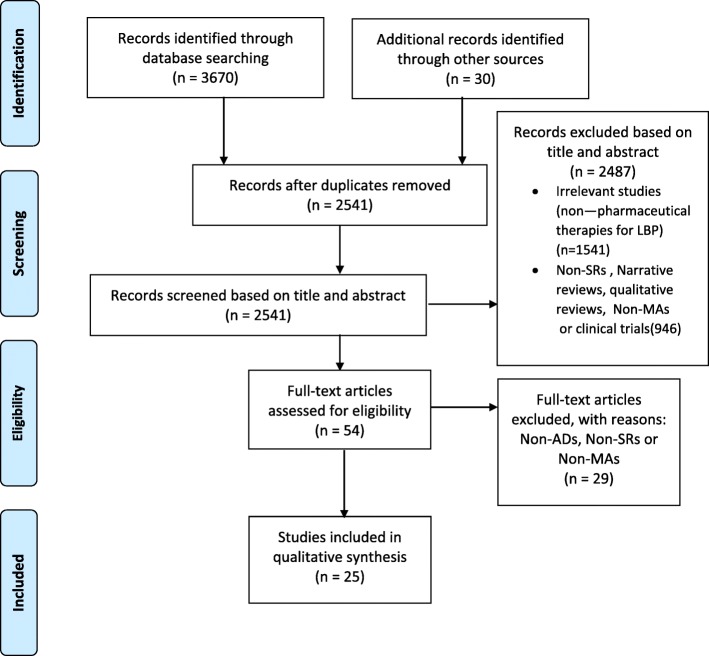
PRISMA Flow Diagram of the Review Search and Identification

Through the initial search, we extracted 3700 potentially relevant articles by searching electronic databases and other resources. After skimming the titles and abstracts and identifying duplications, 3646 articles were excluded. The full texts of the remaining 54 articles were read carefully in their entirety. Twenty-five articles were eligible for the inclusion; 29 Narrative/reviews were excluded from the assessment. All included studies were SRs and MAs on the role of ADs in LBP. The PRISMA flowchart guided the selection process of extracted literature (Fig. 1).

### Characteristics of included SRs

Characteristics of the 25 SRs and MAs are presented in Table 1. Studies were reported between 1992 and 2017. The number of studies included in MAs ranged from 4 to 10 intervention studies on ADs. Studies included were performed on relatively homogeneous patients or populations which suffer from chronic low back pain (CLBP), non-specific low back pain (NLBP), chronic non-specific low back pain (CNLBP) and sciatica. Moreover, multiple AD drug categories with different dosages were considered as intervention. Six out of 25 included studies had no specific subgroups of drug intervention; others consisted of selective serotonin reuptake inhibitors (SSRIs), serotonin and norepinephrine reuptake inhibitors (SNRIs), tricyclic antidepressants (TCAs), tetracyclic antidepressant (TeCA), selective serotonin reuptake inhibitors (SSRIs), and serotonin-norepinephrine reuptake inhibitors (SNRIs). Regarding study design, most studies included in MAs or SRs were randomized controlled trials. In addition, we reported the results of the AMSTAR quality assessment of each study.

### Assessment of methodological quality of included SRs

The assessments of the methodological quality are given in Tables 2 and 3. Out of 25 included studies, 11, 9 and 5 studies were classified as high [2, 14, 16, 17, 33, 34, 37, 41, 44, 45, 48] moderate [12, 35, 36, 42, 43, 47, 49] and low [32, 38–40, 46] quality, respectively.

**Fig. 2:**
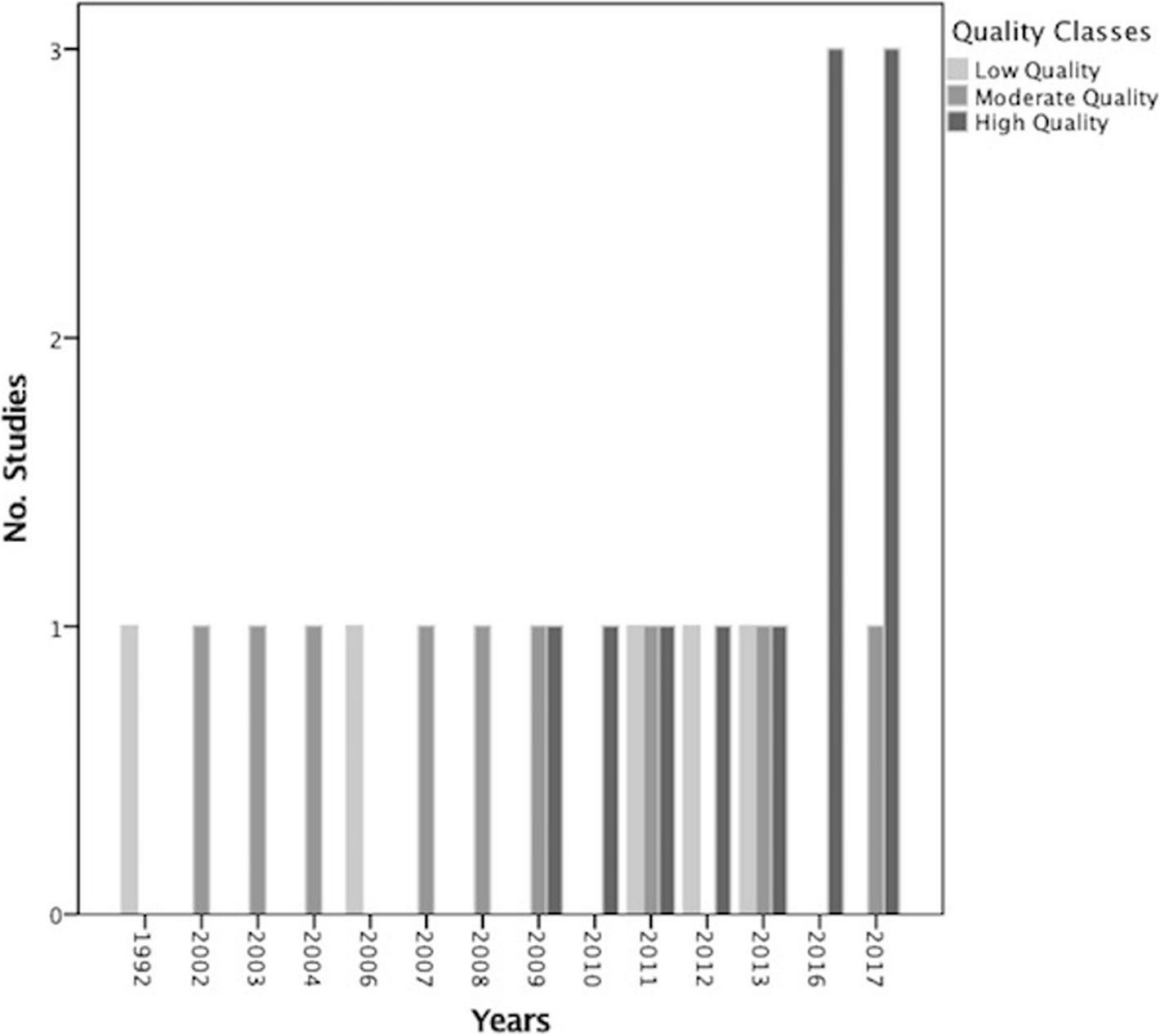
The secular trend of the number and quality of included reviews

Table 3 shows the results of the methodological quality assessment according to each item. Items 1: “Did the research questions and inclusion criteria for the review include the components of PICO (population, intervention, control group and outcome)?”, 3: “Did the review authors explain their selection of the study designs for inclusion in the review?”, 8: “Did the review authors describe the included studies in adequate detail?”, 10: “If meta-analysis (MA) was justified did the review authors use appropriate methods for statistical combination of results?”, 11: “If meta-analysis (MA) was justified did the review authors use appropriate methods for statistical combination of results?” and 16: “Did the review authors report any potential sources of conflict of interest, including any funding they received for conducting the review?” were the most common AMSTAR items in which the studies scored highest, while they lost points in 2: “Did the report of the review contain an explicit statement that the review methods were established prior to conduct of the review and did the report justify any significant deviations from the protocol?” and 15: “If they performed quantitative synthesis did the review authors carry out an adequate investigation of publication bias (small study bias) and discuss its likely impact on the results of the review?”. For items 9, 12, 13 and 14 which were related to the issue of Risk of Bias (RoB) and heterogeneity, they got an average score. 13 (52%) of the studies used a satisfactory technique for assessing the RoB; the Cochrane Collaboration’s tool was the most common tool applied. 5 (50%) of MAs assessed the potential impact of RoB in individual studies on the results of the meta-analysis or other evidence synthesis. 14 (56%) of the review studies accounted for RoB in individual studies when interpreting the results of the review and 16 (64%) of them provided a satisfactory explanation for, and discussion of, any heterogeneity observed in the results of the review. Only 2 (20%) of the meta-analyses out of 10 carried out an adequate investigation of publication bias (small study bias) and discussed its likely impact on the results of the review. Trend analysis showed that since 2016 an increasing trend was observed with regard to the number of publications in this topic with high quality (Fig. 2).

## Discussion

### Methodological quality assessment

To the best of our knowledge, this is the first study to examine specifically the quality of SRs and MAs on the effectiveness of ADs on LBP using AMSTAR 2. In our study, 11 (44%), 9 (36%) and 5 (20%) studies were classified as high, moderate, and low quality, respectively. The former version of AMSTAR assigns even weights to each item and produces an overall score while it is subjected to bias estimation. To overcome this issue, AMSTAR 2 has been designed in a way that it does not estimate an overall score. A high score may disguise critical weaknesses in specific domains, such as an inadequate literature search or a failure to assess the risk of bias (RoB). RoB is a critical section of the appraisal of any systematic reviews. It was conducted by 13 (52%) of the studies which mostly applied the Cochrane Collaboration’s tool. 8 (32%) of the studies assessed quality instead of RoB; we mention them as well to distinguish studies which did none. Contrary to the AMSTAR which focused on the quality assessment of included studies (Item 7), AMSTAR 2 considered RoB in three items [9, 12, 13]. A study may have the highest possible quality and yet have an important risk of bias. For example, in many situations, it is impractical or impossible to blind participants or study personnel to the intervention group. The Newcastle Ottawa Scale, SIGN, and the Mixed Methods Appraisal Tool as well as Cochrane instrument and ROBINS-I are the most comprehensive instruments for assessing RoB. It is important that the impact of RoB be considered in the results of the MAs, so they should assess the impact of this by meta-regression analysis, or by estimating pooled effect sizes by excluding studies at high RoB through sensitivity analysis. 16 (64%) of the included reviews provided a satisfactory explanation for any heterogeneity observed in the results. As a matter of fact, heterogeneity in the SRs and MAs points to the variation in study outcomes between studies. Considering potential sources of heterogeneity which can be related to the domains of bias or PICO description (population, intervention, control group and outcome) is essential. Assessing heterogeneity through Chi-squared test or I-squared index and conducting appropriate methods of analysis like Fixed/Random-effect models and other methods such as meta-regression and sensitivity analysis help detect sources of heterogeneity and strengthen the results. In addition, 2 (20%) of the included MAs carried out an adequate investigation of publication bias and discussed its likely impact on the results. Methods of exploring publication bias in MAs such as funnel plot, Egger and Begg’s test, etc., were presented [50–53]. In addition, the secular trend of studies showed that since 2007 which was the initiation of AMSTAR more publications at moderate to high quality were published and since 2016 most of them were high-quality. It showed that authors were more aware of items which can improve the quality of their research and consequently provide more precise and reliable results.

### Summary of ADs effect on LBP

Most SRs and MAs in this area, illustrated that there was no clear evidence of ADs effectiveness on LPB [2, 16, 34, 41, 54–56] while others achieved contradictory results [18, 35, 36, 57, 58]. Some of them showed that TCAs had significant analgesic effect more than other types of Ads [15, 17, 32, 40, 42, 59–62], while there exists contradiction as well [63]. In addition, some reviews reported a lack of sufficient data for the conclusion [33, 55, 64]. Significant side effects were observed in ADs as well [2, 12, 15, 18].

### Strengths and limitations

The present study is the first to comprehensively assess the methodological quality of SRs on the effect of ADs on LBP. We used the updated version of AMSTAR appraisal tools (AMSTAR 2) which has some merits over the older version. This evaluation can help experts to rely on high-quality studies when getting stuck in the dilemma of conflicting literature. A limitation of our study was that it only included reviews published in English, so publication bias could be introduced.

## Conclusion

Although the trend of publishing high quality papers in ADs effect on LBP increased recently, performing more high-quality SRs and MAs in this field with precise subgroups of the type of pains, the class of drugs and their dosages may give clear and more reliable evidence to help clinicians and policymakers.

## Data Availability

All data generated or analyzed during the current study are included in this published article.

## Abbreviations

ADs: Antidepressants
AMSTAR: Assessment of Multiple Systematic Reviews
CLBP: Chronic Low Back Pain
CNLBP: Chronic Non-specific Low Back Pain
MAs: Meta-Analyses
NLBP: Non-specific Low Back Pain
PICO: Population, Intervention, Control group and Outcome
SNRIs: Serotonin and Norepinephrine Reuptake Inhibitors
SRs: Systematic Reviews
SSRIs: Selective Serotonin Reuptake Inhibitors
TCAs: Tricyclic Antidepressants
TeCA: Tetracyclic Antidepressant
